# Comprehensive analysis of differentially expressed circRNAs and ceRNA regulatory network in porcine skeletal muscle

**DOI:** 10.1186/s12864-021-07645-8

**Published:** 2021-05-01

**Authors:** Meng Li, Na Zhang, Wanfeng Zhang, Wei Hei, Chunbo Cai, Yang Yang, Chang Lu, Pengfei Gao, Xiaohong Guo, Guoqing Cao, Bugao Li

**Affiliations:** College of Animal Science, Shanxi Agricultural University, Taigu, 030801 China

**Keywords:** Pig, Skeletal muscle, Intramuscular fat deposition, DEcircRNA, ceRNA network, circ_0015885

## Abstract

**Background:**

Circular RNA (circRNA), a novel class of non-coding RNA, has a closed-loop structure with important functions in skeletal muscle growth. The purpose of this study was to investigate the role of differentially expressed circRNAs (DEcircRNAs), as well as the DEcircRNA-miRNA-mRNA regulatory network, at different stages of porcine skeletal muscle development. Here, we present a panoramic view of circRNA expression in porcine skeletal muscle from Large White and Mashen pigs at 1, 90, and 180 days of age.

**Results:**

We identified a total of 5819 circRNAs. DEcircRNA analysis at different stages showed 327 DEcircRNAs present in both breeds. DEcircRNA host genes were concentrated predominately in TGF-β, MAPK, FoxO, and other signaling pathways related to skeletal muscle growth and fat deposition. Further prediction showed that 128 DEcircRNAs could bind to 253 miRNAs, while miRNAs could target 945 mRNAs. The constructed ceRNA network plays a vital role in skeletal muscle growth and development, and fat deposition. Circ_0015885/miR-23b/SESN3 in the ceRNA network attracted our attention. miR-23b and SESN3 were found to participate in skeletal muscle growth regulation, also playing an important role in fat deposition. Using convergent and divergent primer amplification, RNase R digestion, and qRT-PCR, circ_0015885, an exonic circRNA derived from *Homer Scaffold Protein 1* (*HOMER1*), was confirmed to be differentially expressed during skeletal muscle growth. In summary, circ_0015885 may further regulate SESN3 expression by interacting with miR-23b to function in skeletal muscle.

**Conclusions:**

This study not only enriched the circRNA library in pigs, but also laid a solid foundation for the screening of key circRNAs during skeletal muscle growth and intramural fat deposition. In addition, circ_0015885/miR-23b/SESN3, a new network regulating skeletal muscle growth and fat deposition, was identified as important for increasing the growth rate of pigs and improving meat quality.

**Supplementary Information:**

The online version contains supplementary material available at 10.1186/s12864-021-07645-8.

## Background

Pig, a key source of animal protein, are widely used in the meat industry where they are an important economic source for animal husbandry. Improving the quality of meat while ensuring growth rate has become both the goal and direction of pig breeding, thereby ensuring that high-quality pork is the first choice for modern people. Skeletal muscle, which accounts for approximately 40% of body weight, is the main meat-producing tissue of pigs. The number and diameter of skeletal muscle fibers are important indicators affecting muscle traits, with pig growth characteristics predominantly reflected in muscle growth and development, thus directly determining meat yield. In addition to muscle fiber type, skeletal muscle intramuscular fat content has important effects on meat tenderness, water holding capacity, flavor, and juiciness [1–3]. Therefore, investigating the molecular mechanisms affecting skeletal muscle growth and development, and fat deposition in pigs is vital to improve the growth rate and meat quality of pigs.

Circular RNA (circRNA), a novel class of non-coding RNA, is characterized by a closed-loop structure generated by pre-mRNA back splicing [4]. Unlike other linear RNAs, circRNAs are covalently closed, thus lacking a 5′ cap and a 3′ tail, both of which typically confer specific properties, including higher stability, RNase R resistance, and longer half-lives [5]. Therefore, circRNA is an ideal biomarker. CircRNAs act in a tissue and developmental stage-specific manner, with numerous studies verifying their important role in skeletal muscle development and intramuscular fat deposition [6–11]. CircRNA has been shown to have multiple biological regulatory functions, the key of which involves adsorbing microRNA (miRNA) and exerting biological functions [12, 13]. CircFGFR4, which acts as an miR-107 sponge, increasing Wnt3a expression, and leading to bovine primary myoblast differentiation, is highly expressed in bovine skeletal muscle [8, 14]. CircLMO7, derived from *LMO7,* can serve as a decoy for miR-378a-3p, resulting in higher HDAC4 expression and decreased MEF2A expression, thereby promoting myoblast differentiation [15, 16]. In addition, circRNA functions in transcriptional and posttranscriptional gene expression regulation, alternative splicing, protein coding, and protein decoy [9, 17–19].

Using the high-throughput sequencing technology and bioinformatics analysis methods, circRNAs have been predicted and identified in humans [12, 20, 21], animals [22], plants [23] and microorganisms [24–26]. Shen et al. [22] sequenced circRNAs in zebrafish (*Danio rerio*), identifying 3868 circRNAs using three algorithms (find_circ, CIRI, and segemehl). Analysis of miRNA target sites on circRNAs shows that some circRNAs may function as miRNA sponges. Lu et al. [23] identified 2354 rice circRNAs using deep sequencing and computational analysis of ssRNA-seq data, of which, 1356 were exonic circRNAs. Huang et al. [27] investigated circRNA expression profiles in the porcine liver of Jinhua and Landrace pigs, identifying 84,864 circRNA candidates in two breeds, with 366 significantly differentially expressed; according to gene ontology analysis, their host genes were found to be involved in lipid biosynthetic and metabolic processes, and were associated with metabolic pathways.

Currently, circRNA research focuses on various diseases, particularly malignant tumors, with circRNA expression in, and effect on, pig muscle development few reported. Here, we selected the Large White (LW) pig, a Western commercial breed, and the Mashen (MS) pig, a Chinese local breed, based on the differences in muscle fiber diameter, density, intramuscular fat content, at different developmental stages (1, 90, and 180 days old) of each pig breeds [28, 29]. RNA sequencing technology and bioinformatics methods were applied to analyze the differential expression of circRNA (DEcircRNA) and its regulatory network during different developmental stages of these two breeds (1, 90, and 180 days old). The present study obtained circRNA expression profiles and differential expression information in pig muscle, also exploring the role of DEcircRNA in muscle development at the omics level. In summary, this study has initiated research into circRNAs role in the muscle development of pigs. The results of this study provide a foundation for research investigating the mechanisms of circRNA in muscle development.

## Results

### Quality control and evaluation of RNA sequencing data

A total of 2,864,087,346 and 2,765,547,488 raw and clean reads, respectively, were obtained from 18 *longissimus dorsi* muscle samples from 1, 90, and 180 days old LW and MS pigs. Following quality control, each sample had a Q20 ≥ 98.17% and a Q30 ≥ 90.25%. Compared with Sscrofa11.1 (http://www.ensembl.org/Sus_scrofa/Info/Index), each sample’s mapping ratio was higher than 77.35% (Additional file 1). In addition, Pearson correlations between the different biological repeats within groups LW1, LW90, LW180, MS1, MS90, and MS180 (LW1, LW90, LW180 represent 1, 90, 180 days of age of LW pigs respectively; MS1, MS90, MS180 represent 1, 90, 180 days of age of MS pigs respectively) were above 0.90 (Additional file 2). Together, these results confirmed that both the samples and sequencing data in this study were reasonable and reliable.

### Identification and confirmation of circRNAs in the *longissimus dorsi* muscle of LW and MS pigs

**Fig. 1:**
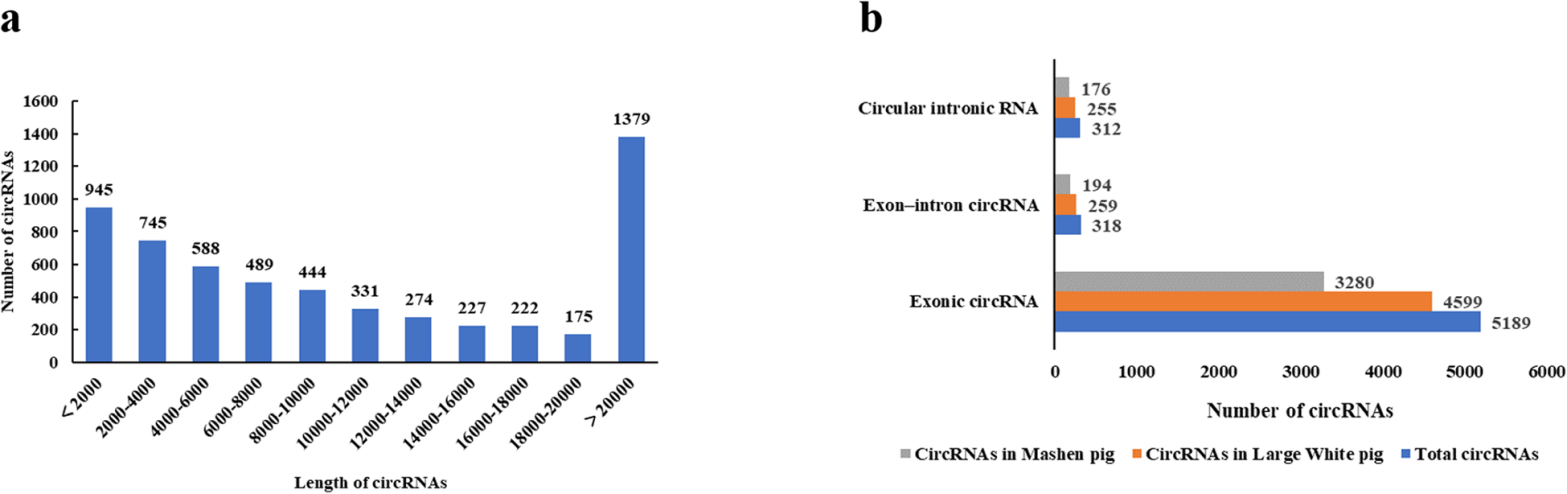
The analysis results of identified circRNAs. **a** Length distribution of circRNAs. **b** Type of circRNAs

Here, a total of 5113 circRNAs were predicted from the *longissimus dorsi* muscle of LW pigs, of which, 3408, 2413, and 3561 circRNAs were predicted in 1, 90, and 180 days old, respectively. A total of 3650 circRNAs were predicted from the *longissimus dorsi* muscle of MS pigs, of which, 1869, 1993, and 2389 circRNAs were predicted in 1, 90, and 180 days old, respectively. A total of 3574 circRNAs existed in both breeds of pigs. As LW and MS pigs have significant genetic differences, the present study predominantly focused on circRNAs common to both breeds, thereby investigating the role of circRNA in skeletal muscle growth and fat deposition in pigs. Additionally, circRNA length predominantly ranged from 100 to 10,000 nt, the shortest and longest of which were 143 and 198,372 nt, respectively (Fig. 1a). As shown in Fig. 1b, among identified circRNAs, the number of exonic circRNAs was the highest, reaching 5189, while the number of intronic circRNA and exon-intron circRNA was 312 and 318, respectively.

### Differential expression of circRNAs at different development stages of the pig *longissimus dorsi* muscle

**Fig. 2:**
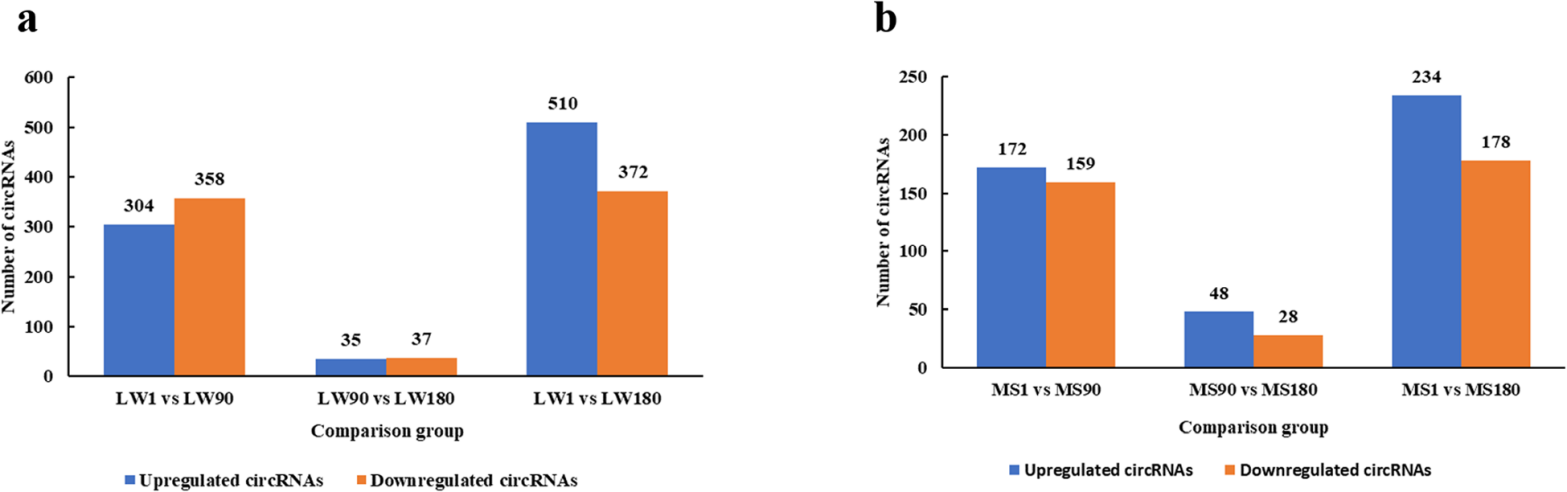
Description of DEcircRNAs at different developmental stages. **a** Upregulated and downregulated circRNA numbers in each LW pig comparison group. **b** Upregulated and downregulated circRNA numbers in each MS pig comparison group

In the LW1 vs LW90 comparison group, there were 662 DEcircRNAs, of which 304 were upregulated and 358 downregulated. In the group LW90 vs LW180, we found 72 DEcircRNAs, of which 35 were upregulated and 37 downregulated. When comparing LW1 and LW180, there were 882 DEcircRNAs, of which 510 were upregulated and 372 downregulated (Fig. 2a). In the MS1 vs MS90 comparison group, there were 331 DEcircRNAs, of which 172 were upregulated and 159 downregulated. In the MS90 vs MS180 group, we found 76 DEcircRNAs, of which 48 were upregulated and 28 downregulated. When comparing MS1 and MS180, we identified 412 DEcircRNAs, of which 234 were upregulated and 178 downregulated (Fig. 2b). Analysis of DEcircRNAs indicated that 539 and 1098 occurred during different MS and LW pig, respectively, developmental stages; 327 were common in both breeds.

### Gene Ontology (GO) and Kyoto Encyclopedia of Genes and Genomes (KEGG) analysis of DEcircRNAs

GO classification results indicated that GO terms annotated via DEcircRNA source genes during different LW and MS pig developmental stages involved three functional classifications: cellular components, biological processes, and molecular functions. In LW pigs, the top three GO terms for cellular component were nuclear parts (103), intracellular membrane-bounded organelle (179), and cytoplasmic parts (220); the top three terms for biological process were phosphorus metabolic process (83), protein metabolic process (136), cellular protein metabolic process (111); the top three terms for molecular function were ATP binding (75), purine ribonucleoside triphosphate binding (92), and nucleotide binding (116). In MS pigs, the top three GO terms for cellular component were organelle part (112), intracellular part (206), and intracellular organelle (123); the top three terms for biological process were cellular protein modification process (52), protein modification process (52), and cellular macromolecule metabolic process (87); the top three terms for molecular function were binding (186), protein binding (108), and nucleotide binding (58). KEGG enrichment analysis showed that DEcircRNAs were mainly enriched in the TGF-β, MAPK, FoxO, Hippo, AMPK, and mTOR signaling pathways, and focal adhesion (Additional file 3). Finally, we screened 44 DEcircRNAs may be related to skeletal muscle growth and intramuscular fat deposition.

### Validation of sequencing data

**Fig. 3:**
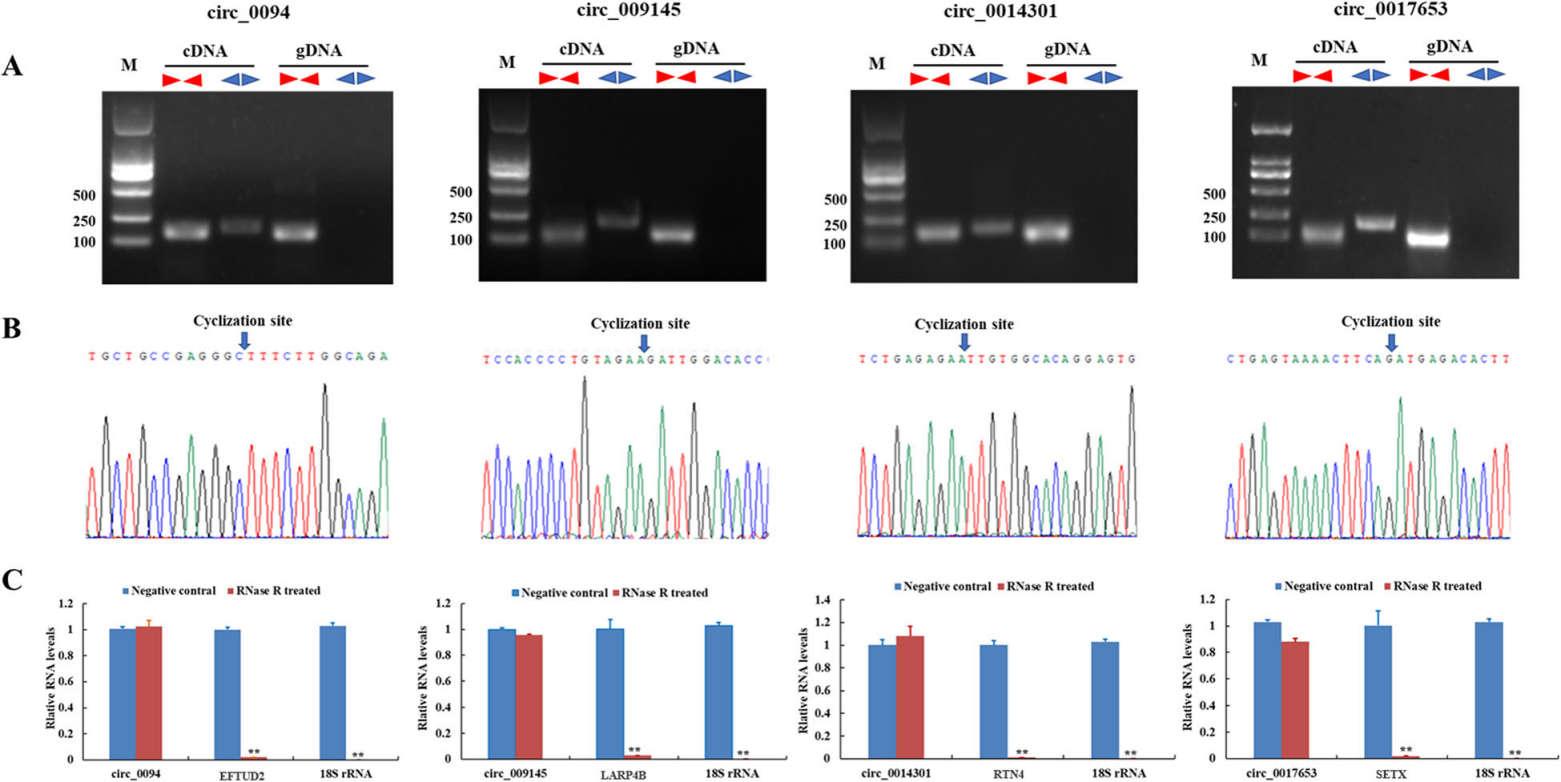
Experimental validation of DEcircRNAs. **a** Divergent and convergent primers amplify circRNA results in cDNA and gDNA samples. **b** Sanger sequencing confirmed the back-splicing junction sequence of circRNAs (Blue arrow points to the splicing site). **c** qRT-PCR results showing the resistance of circRNAs and linear genes to RNase R digestion. Note: Elongation factor Tu GTP binding domain containing 2 (EFTUD2), La ribonucleoprotein 4B (LARP4B), Reticulon 4 (RTN4) and Senataxin (SETX) were the host genes of circ_0094, circ_009145, circ_0017653, and circ_0014301 respectively; * represents significant difference (*P* < 0.05), ** represents extremely significant difference (*P* < 0.01)

**Fig. 4:**
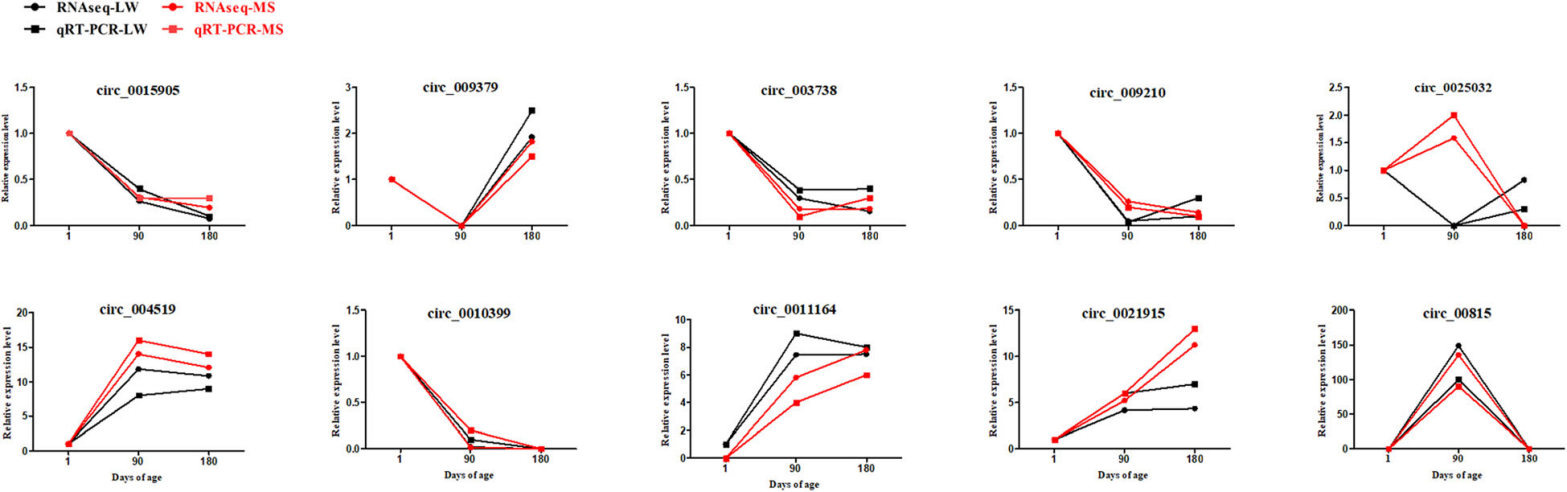
qRT-PCR validation of 10 DEcircRNAs expression levels at different developmental stages

To experimentally confirm candidate pig circRNAs, convergent and divergent primers were designed to amplify each circRNA using both cDNA and genomic DNA (gDNA) as PCR templates. The results showed that convergent primers amplified products from both cDNA and gDNA, while divergent primers amplified circRNAs from cDNA only (Fig. 3a; Additional file 4). Distinct PCR products with the expected size were amplified using convergent and divergent primers; back-splicing sites were verified using Sanger sequencing (Fig. 3b). Additionally, due to its circular structure, circRNA was more resistant to exonuclease RNase R digestion than linear RNA (Fig. 3c). As shown in the Fig. 4, the trend of circRNAs expression between RNAseq and qRT-PCR was similar (Sanger sequencing results of these 10 DEcircRNAs were shown in Additional file 5). These results indicate that predicted pig circRNAs in the current study are credible.

### Construction of a ceRNA network

**Fig. 5:**
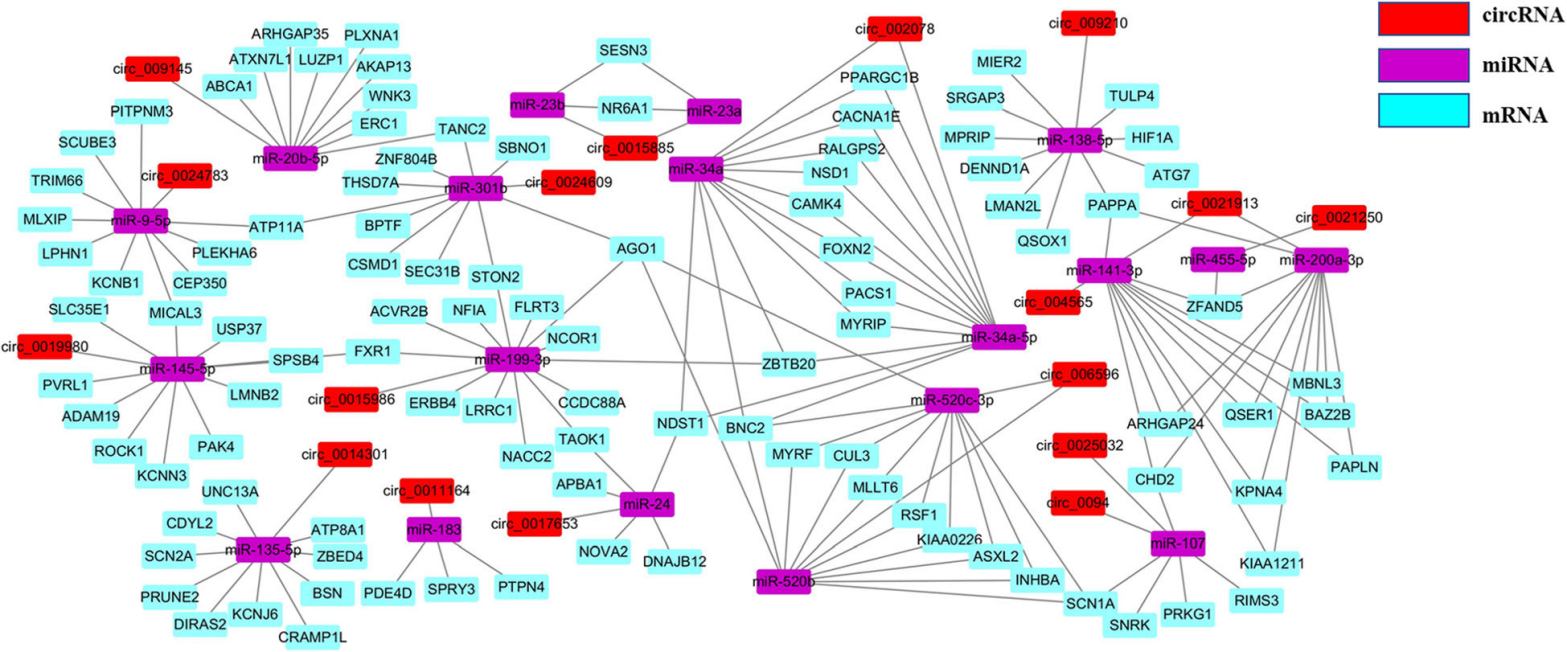
CircRNA/miRNA/mRNA ceRNA network diagram

**Fig. 6:**
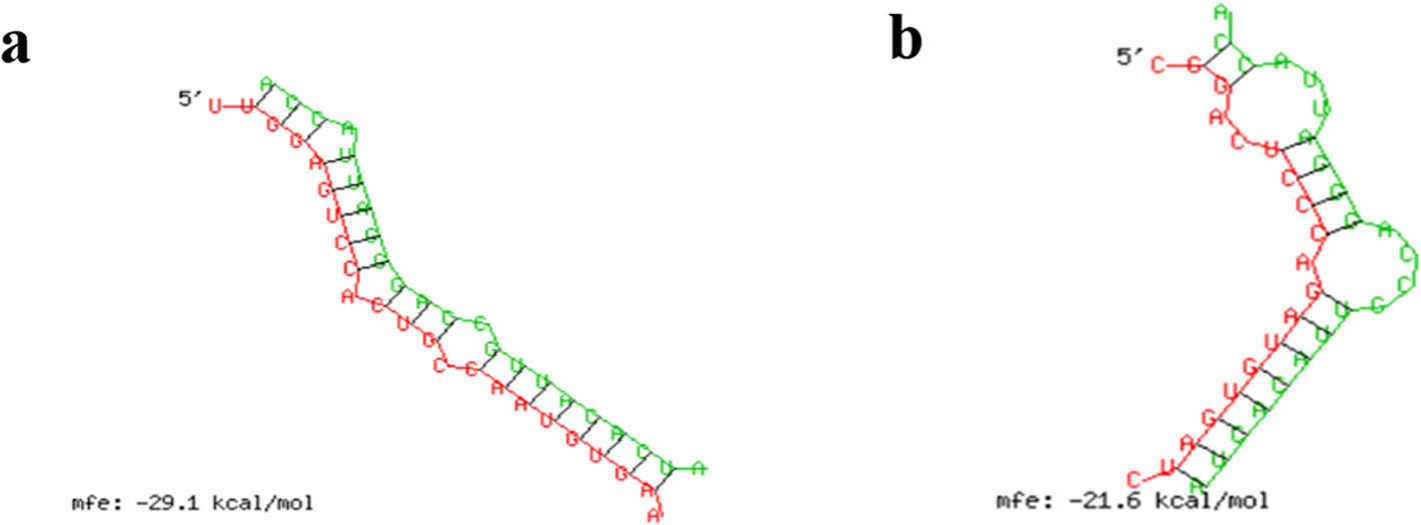
The potential interaction model of circ_0015885/miR-23b/SESN3. **a** The potential interaction model between circ_0015885 and miR-23b from RNAhybrid. **b** The potential interaction model between miR-23b and SESN3 from RNAhybrid

Prediction of 327 DEcircRNAs common at different LW and MS pig growth stages showed that 128 could bind to 253 miRNAs, while miRNAs could target 945 mRNAs. Considering this, we selected circRNAs with relatively high expression levels and muscle or fat functions, according to previous studies, to construct the following ceRNA network diagram (Fig. 5). The constructed ceRNA network plays an important role in skeletal muscle growth and development, and in fat deposition. In this network, circ_0015885/miR-23b/SESN3 attracted our attention. The potential interaction models between circ_0015885 and miR-23b, miR-23b and SESN3 were shown in Fig. 6.

### Experimental validation of circ_0015885

**Fig. 7:**
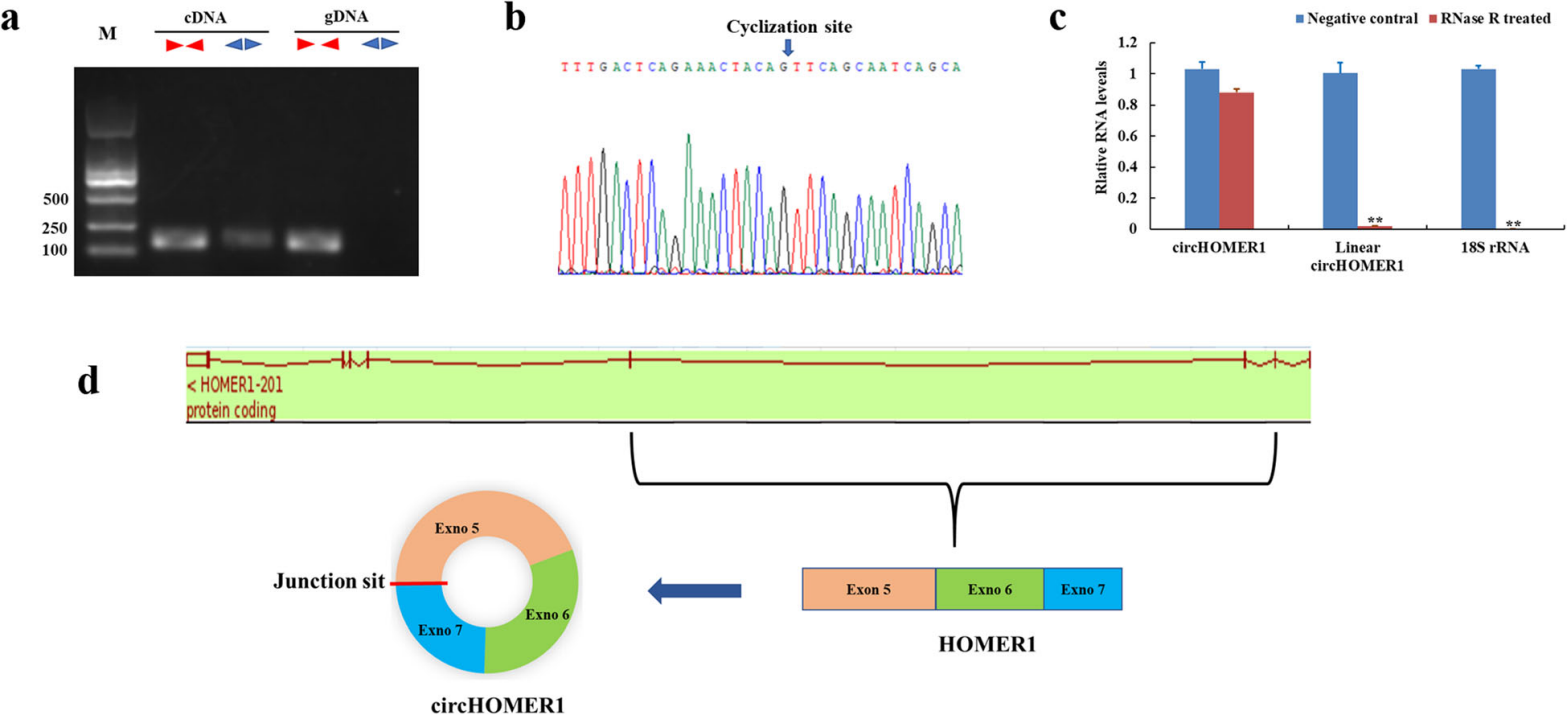
Experimental validation of circ_0015885. **a** Divergent and convergent primers amplify circ_0015885 results in cDNA and gDNA samples. **b** Sanger sequencing confirmed the back-splicing junction sequence of circ_0015885 (Blue arrow points to the splicing site). **c** qRT-PCR results showing the resistance of circ_0015885 and linear genes to RNase R digestion. **d** Schematic diagram of circ_0015885 derived from the *HOMER1* gene. Note: * represents significant difference (*P* < 0.05), ** represents extremely significant difference (*P* < 0.01)

Divergent primers produced a single distinct band in cDNA samples only (Fig. 7a; Additional file 4), indicating that circ_0015885 is a back-splicing product from the pig genome. PCR products from divergent primers were sequenced for junction site verification (Fig. 7b). We treated total RNAs with RNase R treatment, and performed qRT-PCR. The results showed that circ_0015885 was more resistant to RNase R than *Homer Scaffold Protein 1* (*HOMER1)* and *18S rRNA* mRNA (Fig. 7c). Through comparative analysis, we found that circ_0015885 is an exonic circRNA derived from exons 5, 6, and 7 of *HOMER1* (Fig. 7d).

**Fig. 8:**
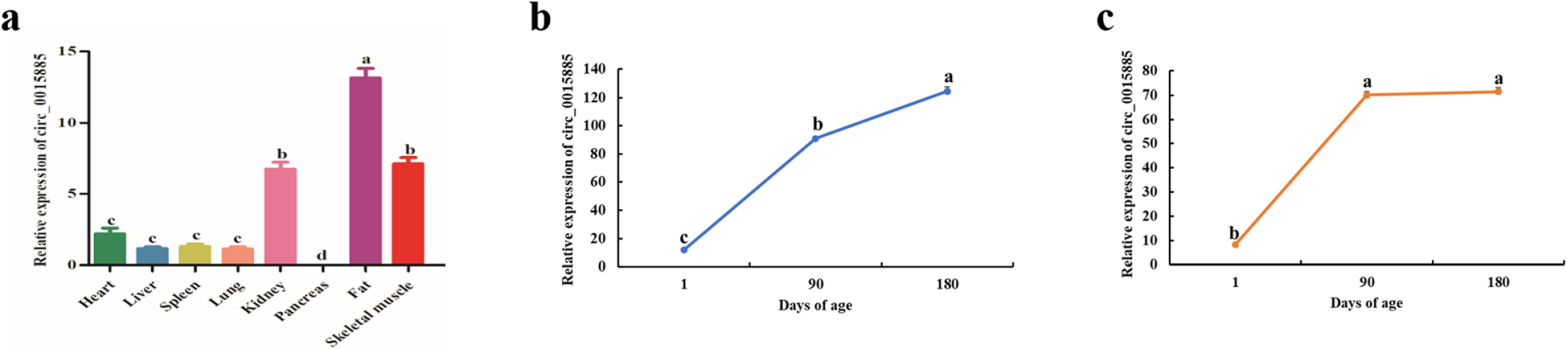
Circ_0015885 expression patterns. **a** Circ_0015885 expression levels in different tissues. **b** Circ_0015885 expression patterns in LW pig skeletal muscle at three developmental stages. **c** Circ_0015885 expression patterns in MS pig skeletal muscle at three developmental stages. Note: Different lowercase letters indicate significant difference, while the same lowercase letters indicate no significant difference

Circ_0015885 expression was detected in different tissues, with the highest expression level found in adipose tissue, followed by skeletal muscle and kidney (Fig. 8a). We also examined the circ_0015885 expression patterns at 1, 90, and 180 days, with results indicating that its expression level was significantly different at different developmental stages (*P* < 0.05) (Fig. 8b and c).

## Discussion

Abundant research in recent years has focused on non-coding RNAs, such as miRNA and long non-coding RNAs, which have important regulatory roles in skeletal muscle growth and development [30]. Recent emerging evidence indicates that circRNAs are another type of non-coding RNA. Through sequencing, bioinformatics and experimental techniques, the present study constructed ceRNA networks related to skeletal muscle development and intramuscular fat deposition, and screened many candidate circRNAs that regulate skeletal muscle development in pigs. CircRNA is a key regulator of skeletal muscle development [31, 32]. Interestingly, many studies have revealed that circRNAs are abundant in skeletal muscle, and that their global expression levels dynamically change during myoblast differentiation [9, 33]. Human circ-ZNF609, derived from ZNF609, shows higher expression in myotubes than in myoblasts, with its siRNA-mediated knockdown reducing myoblast proliferation [9]. Overexpression of circFUT10 inhibits cell proliferation, induces myoblast apoptosis, and enhances myoblast differentiation [34]. CircRNA also plays an important role in fat deposition, with Zhu et al. finding that hsa_circH19 can promote adipogenic differentiation of human adipocytes by targeting PTBP1 [10]. CircRNA_0046367 can remove miR-34a inhibitory action on PPARα, thereby inhibiting liver steatosis [11]. Hence, studying circRNA expression conditions during different porcine skeletal muscle developmental stages, as well as its role in skeletal muscle growth and intramuscular fat deposition in present study, is of great significance.

With the development of high-throughput sequencing technology and bioinformatics analysis methods, increasing numbers of circRNAs have been predicted and identified. Zhang et al. performed deep RNA sequencing of C2C12 myoblasts during cell differentiation, uncovering 37,751 unique circRNAs derived from 6943 host genes. GO analysis showed that many downregulated circRNAs were exclusive to cell division and the cell cycle, while upregulated circRNAs were related to cell development [33]. During embryonic muscle development at 33, 65, and 90 days post-coitus in Duroc pigs, Hong et al. revealed that more than 5000 circRNAs are specifically expressed in embryonic muscle development. Furthermore, they observed that DEcircRNA host genes were enriched in skeletal muscle function during porcine muscle development [35]. Our previous study found significant differences in both growth rate and skeletal muscle growth between LW and MS pigs at different developmental stages (1, 90, and 180 days old) [28, 29, 36]. Fiber diameter significantly increased, while fiber density significantly decreased, with age in both LW and MS pigs. Fiber density had a significant negative correlation with fiber diameter. The myofiber diameter of MS pigs was significantly smaller than that of LW pigs at the same age, while fiber density was much greater than that in LW pigs [28, 29]. At birth, the intramuscular fat content of LW pigs was higher than that of MS pigs. Both at 90 and 180 day old, the intramuscular fat content in the *longissimus dorsi* muscle of MS pigs was higher than that in LW pigs. Considering the differences in skeletal muscle diameter, density, and intramuscular fat content between LW and MS pigs at different developmental stages, examining the role of circRNAs in skeletal muscle growth and intramuscular fat deposition in the *longissimus dorsi* muscle of these two breeds is of great importance. Liang et al. comprehensively analysed circRNAs in nine organs and three skeletal muscles of Guizhou miniature pig and identified 5934 circRNAs [37]. Compared with the study of Liang et al., the present study selected two pig breeds with great genetic differences and three representative stages with significant differences in muscle characteristics and intramuscular fat content, so as to study the role of circRNA in skeletal muscle development in a more comprehensive and representative way. In this study, 1098 and 539 DEcircRNAs were found in the skeletal muscle of LW and MS pigs, respectively, at different developmental stages. Among them, 327 DEcircRNAs co-existed in both breeds, indicating their importance as candidates for regulating skeletal muscle growth and development, as well as intramural fat deposition, in pigs.

CircRNAs can act as miRNA sponges to regulate skeletal muscle growth and fat deposition. In chickens, circRBFOX2 can sponge miR-206, thereby negatively regulating miR-206 expression, increasing *CCND2* (*cyclin D2*) expression, and promoting myoblast proliferation [38–40]. miR-203 has been implicated as a negative regulator of myoblast proliferation and differentiation. CircSVIL acts as a decoy of miR-203, thus playing a positive role in myogenesis [41, 42]. In the present study, ceRNA interaction network analysis demonstrated that circRNAs may be critical regulators of muscle development. Circ_0094 and circ_0025032 were predicted to target miR-107 binding. Li et al. demonstrated that miR-107 inhibited bovine myoblast differentiation, also protecting cells from apoptosis [8]. Wnt3a was identified as a target of miR-107. Knockdown of Wnt3a inhibited bovine myoblast differentiation and apoptosis, an effect similar to that of miR-107 overexpression [8]. Similarly, we predicted that circ_0015986 could be used as a sponge for miR-199a-5p to regulate muscle growth and development. miR-199a-3p regulates C2C12 myoblast differentiation through the IGF-1/AKT/mTOR signaling pathway [43], also regulating smooth muscle cell proliferation and morphology by targeting the Wnt2 signaling pathway [44]. Among the predicted results, many miRNAs related to muscle growth and development, such as miR-135 [45], miR-23b [46], miR-23b [47], and miR-20b-5p [48]. miR-183, miR-23a, and miR-23b may play important roles in porcine skeletal muscle fat deposition. miR-183 significantly accelerates lipid deposition, increasing the expression of fat-forming genes, such as *PPAR*, *C/EBP*, *SREBP-1C*, *FAS*, and *ACC*, by inhibiting *Smad4* mRNA and protein levels [49]. miR-183 can also target LRP6, as well as promote the differentiation and adipogenesis of 3 T3-L1 precursor adipocytes through the Wnt/β-catenin signaling pathway [50]. Through miRNA sequencing, Guan et al. found that miR-23a mediates the formation of bovine skeletal muscle fat. Further studies have shown that miR-23a can reduce lipid accumulation and inhibit PPAR and C/EBPα expression [51]. The above results indicate that circRNAs differentially expressed in the skeletal muscle of LW and MS pigs at different developmental stages play an important role in skeletal muscle growth and development, as well as in intramuscular fat content. Among these, circ_0015885/miR-23b/SESN3 caught our attention.

Circ_0015885, an exonic circRNA, is derived from exons 5–7 of *HOMER1*. Convergent and divergent primer amplification, RNase R digestion, and qPCR confirmed that circ_0015885 is existed, differentially expressed during skeletal muscle development. miR-23b is reportedly involved in many cell functions, including cell proliferation, migration, and differentiation [52, 53]. A recent study showed that miR-23b can inhibit the proliferation of airway smooth muscle cells by targeting Smad3 [47, 54]. The 3′-UTR of SIRT1 mRNA is a direct target of miR-23b, which increases intracellular triglyceride levels by inhibiting SIRT1 expression in HepG2 cells [55]. Sestrins (SESNs), an evolutionarily conserved protein family, are important regulators of metabolic homeostasis. SESNs can maintain metabolic homeostasis by regulating the AMPK-MTOR axis and inhibiting the metabolic syndrome associated with aging and obesity [56, 57]. SESNs belong to the stress protein family and consist of three members, SESN1, SESN2, and SESN3, each of which has specific protein-coding genes [58, 59]. *SESN3*, a stress-sensitive gene, regulates lipid metabolism, directly controlling skeletal muscle fat metabolism. Overexpression of *SESN3* can improve triglyceride accumulation, which is observed to significantly deteriorate following *SESN3* downregulation [60]. *SESN3* mRNA has also been shown to increase significantly during cell differentiation in human primary muscle tubules. SiRNA-mediated silencing of the *SESN3* gene in the muscle tube increases myostatin expression. In type II diabetes, the expression of *SESN3*, which affects skeletal muscle differentiation without changing glycolipid metabolism [61], was increased. Thus, *SESN3* may be involved in the regulation of skeletal muscle growth and lipid deposition. In summary, the circ_0015885/miR-23b/SESN3 network may play a key role in porcine skeletal muscle growth and development, as well as in intramuscular fat deposition.

## Conclusion

Here, a total of 5819 circRNAs were identified in pig skeletal muscle, thereby enriching the porcine circRNA library. In addition, a DEcircRNA-miRNA-mRNA ceRNA network was constructed in which we identified circ_0015885/miR-23b/SESN3, a new network regulating skeletal muscle growth and fat deposition, both of which are important factors for increasing the growth rate of pigs and improving meat quality.

## Methods

All animal experiments in our research were performed in strict accordance with the Code of Ethics of the World Medical Association (http://ec.europa.eu/environment/chemicals/lab_animals/legislation_en.htm). Experimental protocols were approved by the Animal Ethics Committee of Shanxi Agricultural University (Shanxi, China). For this study, a total of nine healthy LW and nine healthy MS male pigs were selected from the Datong Pig Breeding Farm (Shanxi, China). All pigs were kept under the same environmental conditions. Each pig was weaned and castrated at 28 days old. Three pigs each of both breeds were slaughtered at each of the following 3 developmental stages, 1 (early stage), 90 (middle stage), and 180 (later stage) days after birth. All pigs were stunned with electricity to ameliorate the suffering of the pigs before death, followed by exsanguination using transverse incision of the neck. Eight different tissues, including heart, liver, spleen, lung, kidney, pancreas, skeletal muscle, and fat, were collected, snap-frozen in liquid nitrogen, and stored at − 80 °C for further use. The transcriptome of *longissimus dorsi* at each of the 3 developmental stages was subjected to whole transcriptome sequencing analysis.

### Library preparation and sequencing

The cDNA library building process was performed as follows: Total RNA was isolated from muscle tissue samples using TRIzol® Reagent (Invitrogen, USA), according to the manufacturer’s instructions. RNA quantity and purity were checked using a Nanodrop 2000 (Thermo Fisher, USA), while RNA integrity was detected using agarose gel electrophoresis, with the RNA integrity number (RIN) analyzed using an Agilent 2100 Bioanalyzer (Agilent Technologies, USA). For single library preparation, the total amount of RNA should be 5 μg, at a concentration ≥ 250 ng/μL, 1.8 ≤ OD260/280 ≤ 2.2, and a RIN ≥ 7.0. Ribosomal RNAs (rRNAs) were removed from total RNA using a Ribo-Zero Magnetic kit (Epicentre, USA), and fragmented to approximately 200 bp. A TruSeq™ Stranded Total RNA Library Prep Kit (Illumina, USA) was used to prepare the cDNA library. Fragmented RNAs were used to generate first-strand cDNA using random primers. For second-strand synthesis, dTTP was substituted with dUTP in the dNTP reagent, thereby allowing the second base of the cDNA chain to contain A/U/C/G. Following end-repair and A-tailing, 150–200 bp cDNA fragments were isolated, and double-stranded cDNA was ligated to a “Y” adaptor. Single-strand cDNA was then obtained using uracil-N-glycosylase (UNG). Next, PCR amplification was performed to enrich the cDNA libraries. Finally, paired-end sequencing was used in the present study, with sequencing performed on an Illumina HiSeq4000 sequencing platform.

### Quality control for raw reads and circRNA prediction

Quality control for raw reads was determined using SeqPrep (https://github.com/jstjohn/SeqPrep) and Sickle (https://github.com/najoshi/sickle) software. The Q20, Q30, and GC contents of raw reads were calculated. After discarding the reads containing an adapter, reads with over 10% poly-Ns, reads with a mass value of less than 20 nt at the end of the sequence, and sequences whose length was less than 20 bp after mass pruning, remaining clean reads were aligned to the reference pig genome (Sscrofa11.1) using Hisat2 [62] (https://ccb.jhu.edu/software/hisat2/index.shtml).

CIRI [63] software was selected to identify circRNAs for subsequent analysis. CIRI used the BWA-MEM [64] algorithm to perform sequence alignment and find junction reads. Junction reads, which support signals of GT-AG and alternate pairwise shear at the shear site, serve as the basis for circRNA recognition. A dynamic programming algorithm was used to detect circRNA.

### Differential expression analysis of circRNA

Due to the particularity of circRNA, it is difficult to accurately obtain all circRNA reads. Therefore, the expression level of circRNA was estimated via the number of back-spliced reads. In this study, the spliced reads per billion mapping (SRPBM) method was used to estimate the circRNA expression level. The calculation formula was as follows:

$$ \mathrm{SRPBM}=\mathrm{Spliced}\ \mathrm{reads}/\left(\mathrm{Total}\ \mathrm{mapped}\ \mathrm{reads}\right)\times {10}^9. $$

Furthermore, edgeR [65] (http://www.bioconductor.org/packages/2.12/bioc/html/edgeR.html) was used to analyze DEcircRNAs. In this study, the screening criteria for significantly DE circRNAs were FDR < 0.05 and |log2 Fold change (FC)| ≥ 1. According to DEcircRNA SRPBM values, DEcircRNA expression pattern clustering was performed, and the distance calculation algorithm adopted; Spearman’s rank correlation coefficient was used among samples, Pearson correlation coefficient was performed among circRNA, and the method of cluster was hcluster (complete algorithm).

### Enrichment analysis of DEcircRNA host genes

GO analysis of DEcircRNAs was performed using GOATOOLS [66] (https://github.com/tanghaibao/Goatools); GO terms with a corrected *P*-value < 0.05 were considered significantly enriched. KOBAS [67] (http://kobas.cbi.pku.edu.cn/home.do) was used for KEGG pathway enrichment analysis; KEGG pathways with a corrected *P*-value < 0.05 were considered significantly enriched.

### Prediction of DEcircRNA target miRNA and regulatory network construction

TargetFinder [68] and RNAhybrid [69] softwares were used to predict the DEcircRNA miRNA binding target, as well as the DEcircRNA-miRNA target regulatory relationship. Potential loci information of DEcircRNA targeting miRNA was extracted from the results according to the criteria of *P* ≤ 0.05 and free energy ≤35. The target mRNA of miRNA (DEcircRNA targeting miRNA) was further predicted. According to the binding relationship between DEcircRNA and target miRNA, and miRNA and target mRNA, the target regulatory relationship of DEcircRNA-miRNA-mRNA was obtained. Finally, Cytoscape v.3.2.1 software [70] was used to visualize each regulatory network, with default parameters adopted.

### Validation of the sequencing data

To verify the reliability of the RNA sequencing data, four DEcircRNAs (circ_0094, circ_009145, circ_0017653, and circ_0014301) were randomly selected for verification of loop structure, 10 DEcircRNAs were randomly selected to verify expression levels at different developmental stages. Based on NCBI reference sequences, convergent and divergent primers were designed using Primer 5.0 software to validate the existence of these circRNAs. Primer sequences are listed in Additional file 6. All primers used in this study were synthesized by Sangon (Sangon Biotech, Shanghai, China). To confirm the circRNA junction, gDNA and cDNA were used for PCR. All PCR products were sequenced by Sangon Biotech Co., Ltd. Sequence analysis was conducted using DNASTAR software (DNASTAR 7.1, http://www.dnastar.com). For RNase R treatment, 2 μg of total RNA was incubated for 20 min at 37 °C with RNase R (Lucigen Corporation, Wisconsin, USA); this was then used to synthesize cDNA for qPCR. For the control group, the same amount of RNA was incubated for 20 min at 37 °C; this was then used to synthesize cDNA. qRT-PCR was performed using a SYBR PrimeScript™ RT-PCR Kit (Takara) on an ABI-7500 (Life Technologies) under the following conditions: pre-denaturation at 95 °C for 30 s, 45 cycles of 95 °C for 5 s and 60 °C for 34 s, one cycle of 95 °C for 15 s, 60 °C for 1 min, and 95 °C for 30 s. All qRT-PCR reactions for each gene were performed using three biological replicates, with three replicates per experiment. *18S rRNA* was used as the internal gene. The 2^–ΔΔCt^ method was used to calculate fold changes in circRNA expression. Statistical differences among different time points were identified using ANOVA. Data were expressed as “means ± SEM”. *P* < 0.05 indicates the difference is significant. *P* < 0.01 represents extremely significant difference.

### Validation of circ_0015885

Convergent and divergent primer amplification, RNase R digestion, and qRT-PCR were used to determine the existence of circ_0015885. The circ_0015885 expression pattern at different skeletal muscle developmental stages, as well as the expression profile in various pig tissues, were detected using qRT-PCR.

### Availability of data and materials

The sequencing data were deposited in the Sequence Read Archive with the accession number SRP068558 (https://trace.ncbi.nlm.nih.gov/Traces/sra_sub/sub.cgi?login=pda).

## Supplementary Information

**Additional file 1: Table S1.** Number of RNA-sequencing Reads and Mapping Results.

**Additional file 2: Table S2.** Pearson correlations between different biological repeats within each LW and MS pigs’ *longissimus dorsi* muscle.

**Additional file 3: Figure S1.** GO and KEGG results of DEcircRNAs in LW and MS pigs. (a) and (b) are GO and KEGG results of LW pigs; (c) and (d) are GO and KEGG results of MS pigs.

**Additional file 4: Figure S2–6.** Original gel images. Figure S2–5 correspond to the Fig. 3a, Figure S6 corresponds to the Fig. 7a.

**Additional file 5: Figure S7.** Sanger sequencing results of DEcircRNAs.

**Additional file 6: Table S3.** Primers used in this study.

## Data Availability

Not applicable.

## Abbreviations

LW: Large White pig
MS: Mashen pig
circRNA: Circular RNA
miRNA: MicroRNA
DEcircRNA: Differentially expressed circular RNA
GO: Gene ontology
KEGG: Kyoto Encyclopedia of Genes and Genomes
gDNA: Genomic DNA
EFTUD2: Elongation factor Tu GTP binding domain containing 2
LARP4B: La ribonucleoprotein 4B
RTN4: Reticulon 4
SETX: Senataxin
HOMER1: Homer Scaffold Protein 1
CCND2: Cyclin D2
SESNs: Sestrins
RIN: RNA integrity number
UNG: Uracil-N-glycosylase
SRPBM: Spliced reads per billion mapping method
FC: Fold change
