# Supplementary material for: Comprehensive analysis of differentially expressed circRNAs and ceRNA regulatory network in porcine skeletal muscle
Source: BMC Genomics. 2021 May 1;22:320. doi: 10.1186/s12864-021-07645-8 (PMC8088698; doi:10.1186/s12864-021-07645-8)
Supplement: Supplementary file 1 — Additional file 1: Table S1. Number of RNA-sequencing Reads and Mapping Results. [file 12864_2021_7645_MOESM1_ESM.docx]

**Table S1 Number of RNA-sequencing Reads and Mapping Results**

| Sample | Raw Reads | Clean Reads | Mapped Reads | Mapping Ratio | Q20% | Q30% | GC% |
| --- | --- | --- | --- | --- | --- | --- | --- |
| LW1-1 | 131619798 | 127223042 | 123964536 | 97.44% | 98.36 | 91.18 | 44.60 |
| LW1-2 | 178972530 | 172594644 | 165390249 | 95.83% | 98.32 | 91.02 | 45.07 |
| LW1-3 | 235040700 | 227483852 | 216145572 | 95.02% | 98.34 | 91.02 | 45.43 |
| LW90-1 | 139057078 | 134005680 | 131939436 | 98.46% | 98.28 | 90.75 | 47.06 |
| LW90-2 | 134133150 | 129710492 | 127485459 | 98.28% | 98.34 | 91.03 | 45.97 |
| LW90-3 | 220457948 | 213032226 | 206237088 | 96.81% | 98.31 | 90.92 | 46.50 |
| LW180-1 | 174125908 | 167684282 | 162160216 | 96.71% | 98.22 | 90.54 | 46.48 |
| LW180-2 | 165542866 | 159553752 | 123421558 | 77.35% | 98.17 | 90.25 | 47.92 |
| LW180-3 | 193717042 | 186820488 | 162933690 | 87.21% | 98.21 | 90.45 | 47.07 |
| MS1-1 | 114207032 | 110538984 | 107774908 | 97.50% | 98.36 | 91.07 | 45.45 |
| MS1-2 | 152361170 | 147296894 | 143276850 | 97.27% | 98.38 | 91.18 | 45.30 |
| MS1-3 | 151920970 | 146867584 | 143433933 | 97.66% | 98.37 | 91.13 | 45.42 |
| MS90-1 | 116498178 | 112220514 | 109633243 | 97.69% | 98.25 | 90.61 | 47.17 |
| MS90-2 | 155940498 | 150861352 | 147606416 | 97.84% | 98.36 | 91.08 | 46.30 |
| MS90-3 | 147834156 | 142634632 | 134311334 | 94.16% | 98.28 | 90.78 | 46.79 |
| MS180-1 | 142125072 | 137132258 | 134405348 | 98.01% | 98.30 | 90.85 | 46.31 |
| MS180-2 | 136944902 | 132207018 | 129560199 | 98.00% | 98.36 | 91.09 | 45.74 |
| MS180-3 | 173588348 | 167679794 | 164563712 | 98.14% | 98.34 | 91.05 | 45.49 |
