# Supplementary material for: Comprehensive analysis of differentially expressed circRNAs and ceRNA regulatory network in porcine skeletal muscle
Source: BMC Genomics. 2021 May 1;22:320. doi: 10.1186/s12864-021-07645-8 (PMC8088698; doi:10.1186/s12864-021-07645-8)
Supplement: Supplementary file 3 — Additional file 3: Figure S1. GO and KEGG results of DEcircRNAs in LW and MS pigs. (a) and (b) are GO and KEGG results of LW pigs; (c) and (d) are GO and KEGG results of MS pigs. [file 12864_2021_7645_MOESM3_ESM.docx]

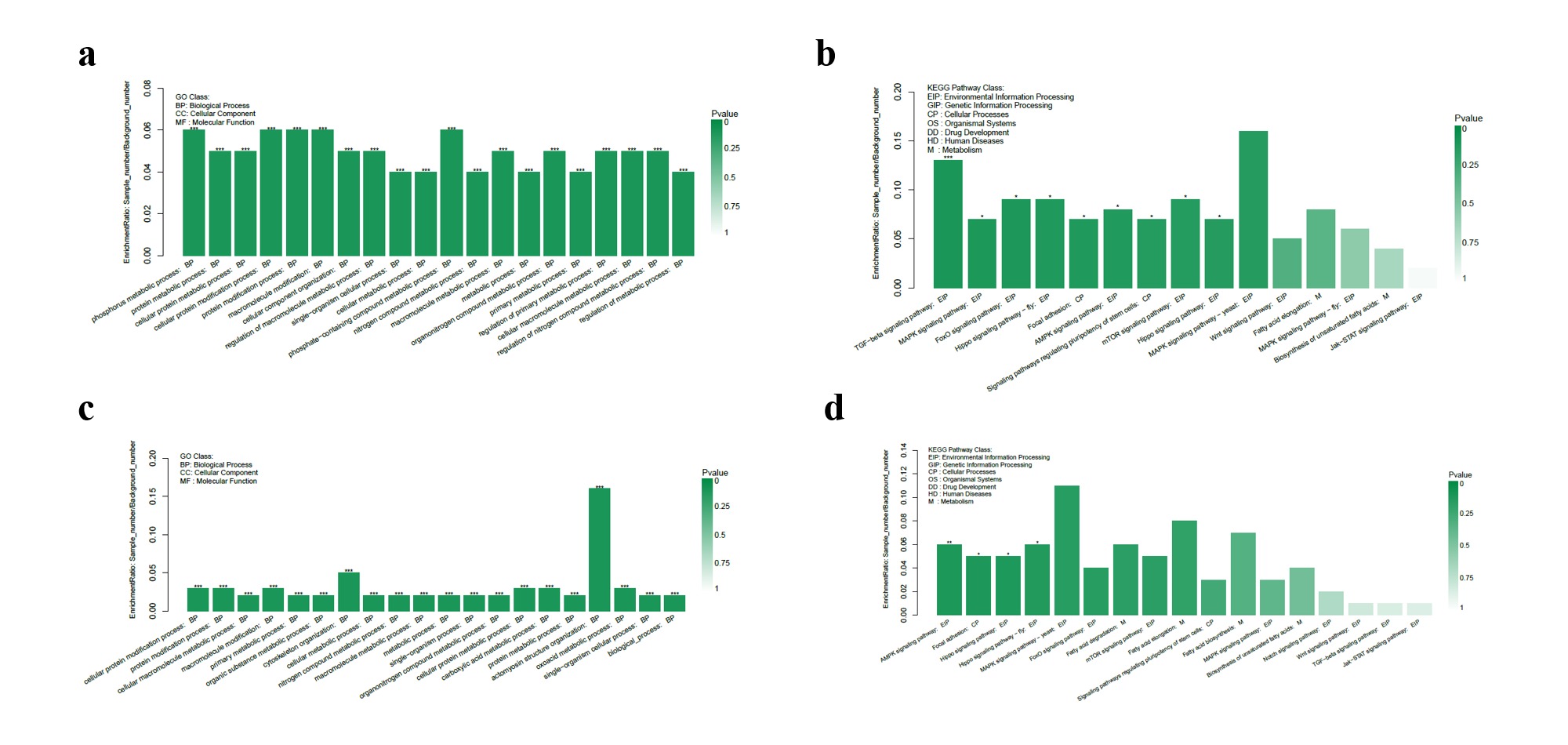
**Figure S1 GO and KEGG results of DEcircRNAs in LW and MS pigs. (a) and (b) are GO and KEGG results of LW pigs; (c) and (d) are GO and KEGG results of MS pigs.**
