# Supplementary material for: Comprehensive analysis of differentially expressed circRNAs and ceRNA regulatory network in porcine skeletal muscle
Source: BMC Genomics. 2021 May 1;22:320. doi: 10.1186/s12864-021-07645-8 (PMC8088698; doi:10.1186/s12864-021-07645-8)
Supplement: Supplementary file 4 — Additional file 4: Figure S2–6. Original gel images. Figure S2–5 correspond to the Fig. 3a, Figure S6 corresponds to the Fig. 7a. [file 12864_2021_7645_MOESM4_ESM.docx]

Original Figures


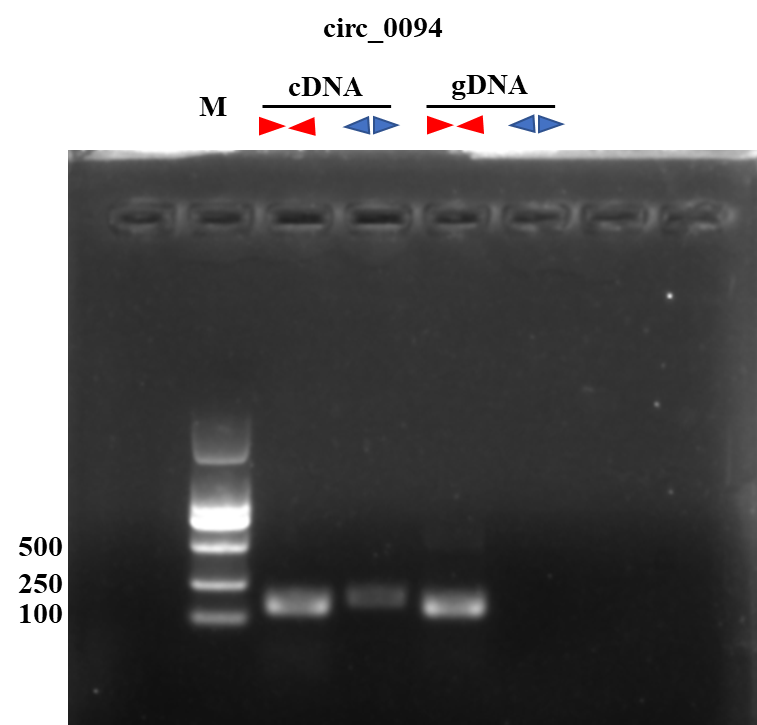


Figure S2 Divergent and convergent primers amplify circ_0094 results in cDNA and gDNA samples.


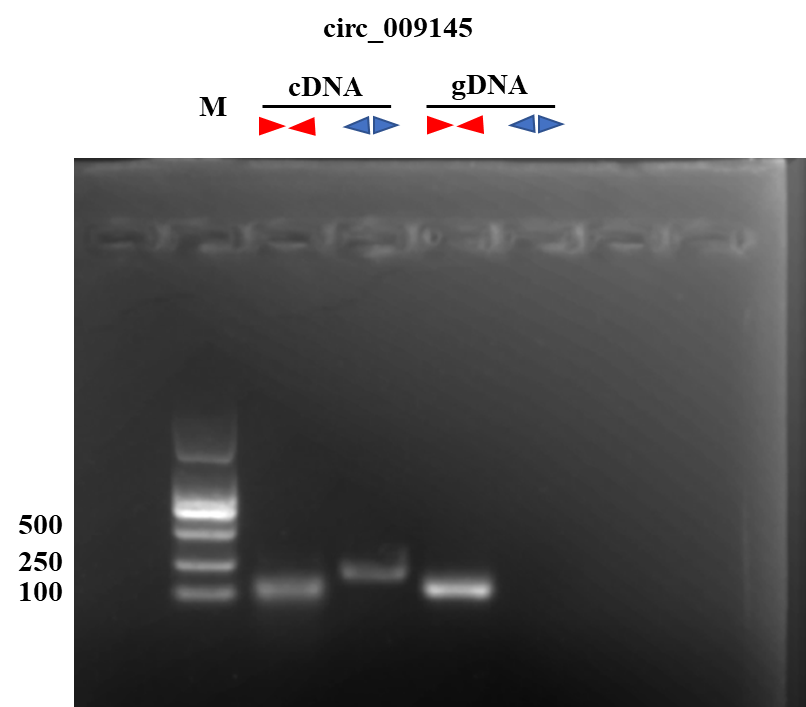


Figure S3 Divergent and convergent primers amplify circ_009145 results in cDNA and gDNA samples.


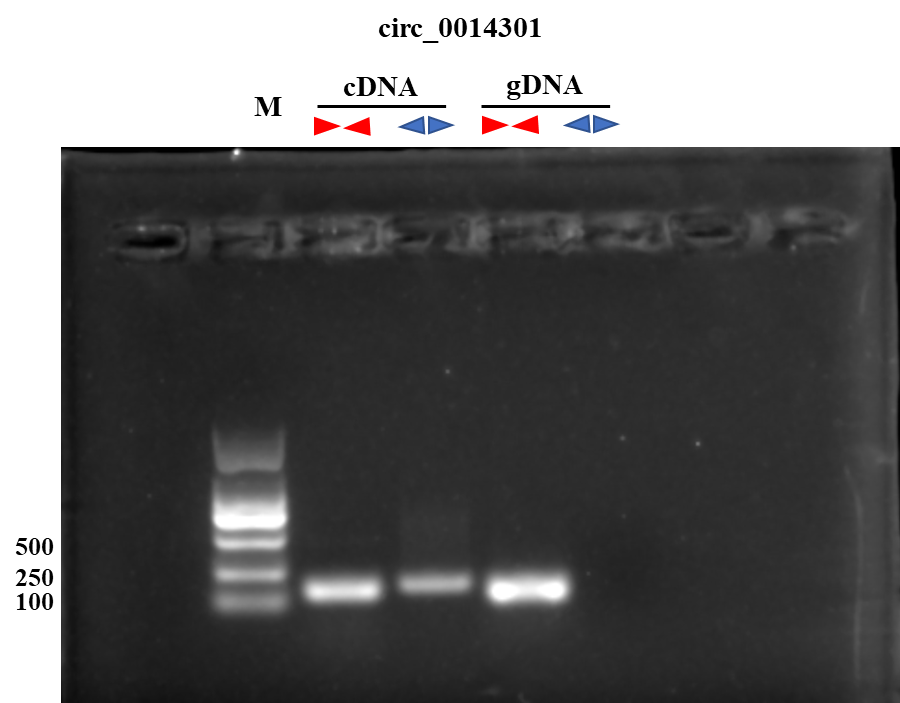


Figure S4 Divergent and convergent primers amplify circ_0014301 results in cDNA and gDNA samples.


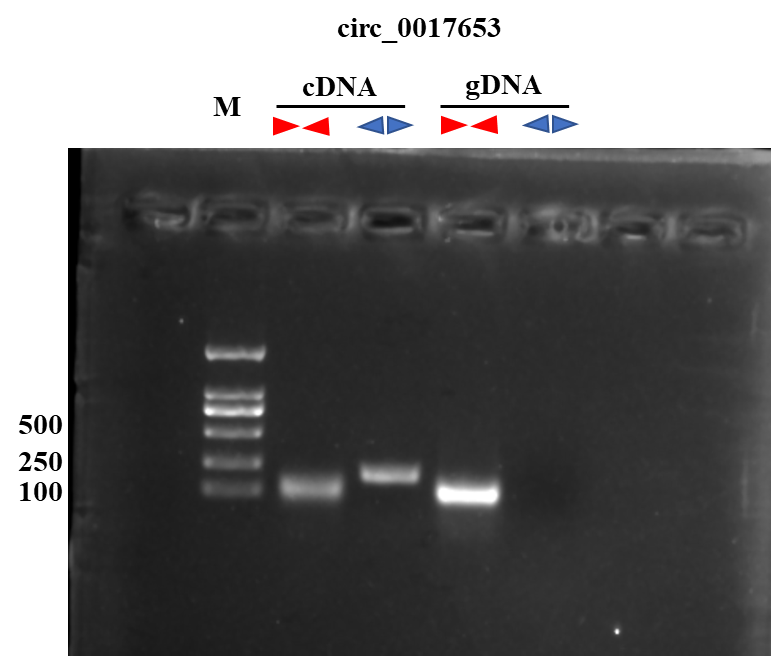


Figure S5 Divergent and convergent primers amplify circ_0017653 results in cDNA and gDNA samples.


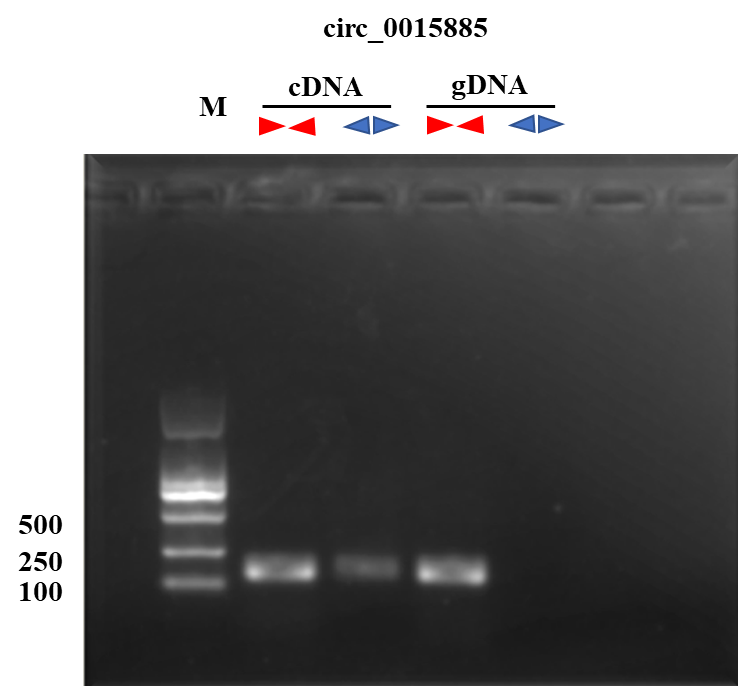


Figure S6 Divergent and convergent primers amplify circ_0015885 results in cDNA and gDNA samples.
