# Supplementary material for: Comprehensive analysis of differentially expressed circRNAs and ceRNA regulatory network in porcine skeletal muscle
Source: BMC Genomics. 2021 May 1;22:320. doi: 10.1186/s12864-021-07645-8 (PMC8088698; doi:10.1186/s12864-021-07645-8)
Supplement: Supplementary file 6 — Additional file 6: Table S3. Primers used in this study. [file 12864_2021_7645_MOESM6_ESM.docx]

**Table S3 Primers used in this study**

| **Name** | **Primer sequence (5’→3’)** | | **Tm (℃)** | **Product Size (bp)** |
| --- | --- | --- | --- | --- |
| circ_0015885 | convergent primer | F: ACATTGGGAGGCTGAACTGG  R: GCAGACGTTCTGCTTCCTCT | 60 | 134 |
|  | divergent primer | F: TGGAAGAGACGCTGAAAGTGA  R: AGCCTCCCAATGTTTGCTGA | 55 | 138 |
| circ_0094 | convergent primer | F: GAGCAGACTCACCCGGAAAT  R: TCTGGCAAGACCACTGTCAC | 60 | 125 |
|  | divergent primer | F: TCTCAGATGGAGTGGTCCTGT  R: TTTCCGGGTGAGTCTGCTCT | 60 | 160 |
| circ_009145 | convergent primer | F: AGAAGTCCCGAGAATGCAGC  R: ATTTTCCGGCGGTGACTCAT | 60 | 105 |
|  | divergent primer | F: GGCCAAATCAGAATCGTTGC  R: TTCCGGCGGTGACTCATACT | 59 | 190 |
| circ_0014301 | convergent primer | F: TGCGCAGTGGGAAATCTGAG  R: TTGCGTGGTTACCAGAGAGC | 60 | 113 |
|  | divergent primer | F: GCTCTCTGGTAACCACGCAA  R: GAGCTGATCCACTCCTGTGC | 60 | 163 |
| circ_0017653 | convergent primer | F: ACTCCTCTGCAGGCAAGTTC  R: ACTATGTTAATGGGCACAGAGT | 60 | 98 |
|  | divergent primer | F: TTTGGCTGCTCCAACAGAGAT  R: TGTATCACAGGCTCAGATGCAG | 60 | 189 |
| circ_0015905 | divergent primer | F: AGGAAACCGGAAACCTGGTG  R: ACCGTGTTGGGTATGCTTGT | 60 | 165 |
| circ_009379 | divergent primer | F: CCCTGAATGAGACGGTGGTC  R: GTGCTCCTCAGGTTGGTCAT | 60 | 199 |
| circ_003738 | divergent primer | F: TTGGCTACTTCGTGCAGCTA  R: TCCTTATGTGACTGTCGGCTG | 60 | 171 |
| circ_009210 | divergent primer | F: ACAAGAAAGCCAGCTTCGTAA  R: TGCCATCACCATCTCAGCAT | 59 | 174 |
| circ_0025032 | divergent primer | F: GAGAACCAAGCTCAGCAGTTG  R: CGCCCGGTTTAGCTATCTCC | 60 | 109 |
| circ_004519 | divergent primer | F: AACATGACCGTGGCACAAGA  R: CTGGCCCTTGTCGGGTTTC | 60 | 129 |
| circ_0010399 | divergent primer | F: ATCCTGTTCCACCCTCCTGA  R: TGGCACAAGGATCAGCCAAT | 60 | 162 |
| circ_0011164 | divergent primer | F: ATCCATTGATAATATGG  R: ATGTGAACGCATGGCACTTG | 59 | 294 |
| circ_0021915 | divergent primer | F: GTGCCCAGTTCTGGCAACA  R: CCTCTGCCACTTTGTTTCTTCTT | 60 | 148 |
| circ_00815 | divergent primer | F: GGTCAAGAAGGAGGACACGG  R: CAGGTCGAACAGCTTCTGGT | 60 | 148 |
| *18S rRNA* |  | F: CCCACGGAATCGAGAAAGAG  R: TTGACGGAAGGGCACCA | 60 | 132 |

Note: When using gDNA as template, the forward primer sequence of circ_0094 is F: ACCGTGTGCATCAACAAGATT R: TCAATCCATTGACCTCATCTACG. The product size was 106 bp and Tm was 60℃.
